# Long-acting Paliperidone Palmitate treatment in an Ekbom’s Syndrome secondary to Lewy Body Dementia: A case report

**DOI:** 10.1192/j.eurpsy.2022.1211

**Published:** 2022-09-01

**Authors:** L. Orsolini, D. Corona, M. Giordani, V. Salvi, U. Volpe

**Affiliations:** Unit of Clinical Psychiatry, Department Of Neurosciences/dimsc, Polytechnic University Of Marche, Ancona, Italy

**Keywords:** Ekbom delusion paliperidone Lewy

## Abstract

**Introduction:**

Ekbom Syndrome (ES) is a condition characterized by the fixed, delusional belief that one’s body is infested by parasites or other vermin, in absence of supporting clinical evidence. Literature suggests antipsychotic treatment for the management of behavioural and psychotic symptomatology, long-acting-injectable (LAI) antipsychotics for poor compliance.

**Objectives:**

A case report of a 70-year-old woman with an ES diagnosis treated with LAI palmitate paliperidone was followed-up for an 8-months period, resulting in a confirmed diagnosis of secondary ES to Lewy Body Dementia (LBD).

**Methods:**

Patient was admitted to the local psychiatry ward presenting with new-onset visual and tactile hallucinations and social withdrawal. She was diagnosed with ES in a context of executive functions impairment. She was initially treated with risperidone, then switched to LAI Paliperidone due to poor compliance. Subsequently she was monitored monthly for 8 months by administering PANSS, MOCA, GAF, BPRS, PSP, complete neurocognitive assessment and neuroimaging studies

**Results:**

After 8 months a progressive cognitive deterioration and worsening of motor impairment confirmed a secondary ES to a LBD. Meanwhile, a significant reduction of psychotic symptomatology (delusions and somatic hallucinations) was observed at BPRS and PANSS positive scale, even after treatment discontinuation due to the onset of extrapyramidal symptoms of the underlying condition.

**Conclusions:**

LAI Paliperidone treatment induced a complete remission of psychotic symptoms, with no relapse even after discontinuation of treatment. Moreover, close observation during follow-up allowed early diagnosis of LBD, which has been associated with a more favorable course.

**Disclosure:**

No significant relationships.

